# Umbilical artery tone in maternal obesity

**DOI:** 10.1186/1477-7827-7-6

**Published:** 2009-01-22

**Authors:** Mark P Hehir, Audrey T Moynihan, Siobhan V Glavey, John J Morrison

**Affiliations:** 1 Department of Obstetrics and Gynaecology, Clinical Science Institute, University College Hospital Galway, Newcastle Road, Galway, Ireland

## Abstract

**Background:**

The increasing prevalence of obesity constitutes a major health problem in obstetrics with implications for feto-maternal growth and wellbeing. This study investigated and compared the contractile properties of umbilical arteries excised from obese women, with those excised from women with a normal body mass index (BMI).

**Methods:**

Sections of umbilical artery were obtained from umbilical cord samples immediately after delivery and mounted for isometric recording in organ tissue baths under physiological conditions. Cumulative additions of 5-Hydroxytryptamine (5-HT) and Prostaglandin F-2alpha (PgF2alpha) were added in the concentration range of 1 nmol/L to 10 micromol/L. Control vessels were exposed to Krebs physiological salt solution (PSS) only. The resultant effects of each drug addition were measured using the Powerlab hardware unit.

**Results:**

5-HT exerted a significant effect on human umbilical artery tone at concentrations of 100 nmol/L, 1 micromol/L, and 10 micromol/L in normal (n = 5; P < 0.05) and obese (n = 5; P < 0.05) women. The contractile effect was significantly greater in vessels from obese women {Mean Maximum Tension (MMT) = 4.2532 g} than in those from women of normal BMI (MMT = 2.97 g; P < 0.05). PgF2alpha exerted a significant contractile effect on vessels at 1 micromol/L and 10 micromol/L concentrations when compared with controls (n = 5; P < 0.05). There was a non-significant trend towards an enhanced tone response in vessels from obese women (MMT = 3.02 g; n = 5), in comparison to vessels from women of a normal BMI (MMT = 2.358 g; n = 5; P > 0.05).

**Conclusion:**

These findings support the hypothesis that endogenous regulation of umbilical artery tone is altered in association with maternal obesity. This may be linked to the cardiovascular effects of secretory products of adipose tissue, with implications for the feto-maternal circulation.

## Background

It has become evident in recent years that there is a marked increase in the prevalence of obesity among the adult and child population [1,2]. While some of the adverse effects of this on health and wellbeing are obvious, the more subtle systemic effects of the so called metabolic syndrome, are hitherto not fully understood. In obstetric practice, it is well established that obesity is associated with a wide range of disorders in pregnancy including hypertensive disease, impaired glucose tolerance, a greater need for caesarean section, problems with labour and delivery, and fetal macrosomia [3-5], and it has recently been indicated that secretory products of adipose tissue may modulate uterine activity[6,7]. While it is clear that obese women are much more likely to deliver large for dates infants, the potential metabolic implications of this for the mother or baby are unknown. What is also not known is whether obesity may exert a metabolic modulation on the feto-placental vasculature, and hence have implications for fetal growth and wellbeing during pregnancy.

It is well established for adult cardiovascular regulation, that obesity may have an adverse effect in terms of vascular stiffness and its effects on arterial dynamics[8]. The potential reasons behind this are complex, but it is postulated to be likely that sympathetic over-activity and metabolic parameters play a major role. In addition, there may be variations in endothelial function and alterations in the coagulation process, which may give rise to arterial dysfunction in association with obesity[9,10]. There are no data to our knowledge in relation to the feto placental vessels and their regulation in obese women. We hypothesised that there may be dysfunction, or altered contractile properties, in feto-placental vessels in association with maternal obesity. The aim of this study was to investigate the response to standard vasoactive agents of umbilical artery preparations excised from the cord relating to the fetus delivered by obese women, and compare the findings to those observed in preparations from women of a normal Body Mass Index (BMI) category.

## Methods

### Tissue collection

Umbilical artery samples were obtained from 28 women after delivery at term, a number of samples (n = 8) were not included due to unsuitability for reasons such as failure to contract, tissue death and poor sample quality or size. All deliveries were either normal vaginal deliveries(20), or elective cesarean deliveries(8), and all were uncomplicated. The median gestation at delivery was 39 weeks. The reasons for cesarean delivery included previous cesarean section (7) and presumed cephalo-pelvic disproportion (1). The median parity value of the women at the time of delivery was 1 (range 0–3). There was no evidence of hypertensive disease in any of the subjects. The mean BMI of those in the normal group was 24.02 kg/m^2^, and that of the obese cohort found was 39.55 kg/m^2^.

A BMI of 19.8 – 25.9 kg/m^2^was regarded as normal for patient recruitment, and obesity was classified as a BMI greater than 35 kg/m^2^. Sections of umbilical cord approximately 10 cm in length were excised from the proximal segment of cord (i.e. closest to the placental attachment) immediately after delivery, from both groups of women. Samples were immediately placed in cold buffered Krebs-Henseleit physiological salt solution (pH 7.4), in order to mimic normal physiological conditions. The physiological salt solution (PSS) was of the following composition: potassium chloride 4.7 mmol/L, sodium chloride 118 mmol/L, magnesium sulphate 1.2 mmol/L, calcium chloride 1.2 mmol/L, potassium phosphate 1.2 mmol/L, sodium bicarbonate 25 mmol/L and glucose 11 mmol/L. Samples were stored at 4°C for a maximum of one hour before experiments took place as this ensured that samples retained a certain amount of intrinsic tone. Samples used after this time did usually not exhibit contraction when exposed to agonists. Research Ethics Committee Approval at University College Hospital Galway was obtained prior to the design of the experiment and written informed consent of every patient was obtained prior to taking samples.

### Tissue bath experiments

Umbilical arteries were carefully dissected from the section of umbilical cord by removal of the surrounding Wharton's Jelly. Vessels were cut in rings each measuring 4 – 5 mm in axial length. Rings were then suspended individually on stainless steel hooks inserted into their lumens and mounted under 2 g (30 mN) of isometric tension from a force-reading transducer, in glass-jacketed tissue baths as previously described [11.12]. Each bath contained 10 ml of Krebs PSS, at pH 7.4, at 37°C, and gassed continuously with a mixture of 95% oxygen/5% carbon dioxide. Rings were allowed to equilibrate for a 90 minute period, with regular washouts of PSS at 20 minute intervals. During this time spontaneous tone developed as the vessels returned to a state that mimicked normal physiological conditions.

After the equilibration period, the vessel rings were challenged with 60 mM Potassium Chloride (KCl). A washout with PSS was carried out once the maximum response had reached a plateau, and a 20 minute recovery period was allowed to allow the contraction of the vessel to return to baseline again. The KCl challenge was performed three times. After the last KCl challenge, a 40 minute recovery period was allowed.

An agonist (5-HT or PgF2_α_) was then added to the tissue bath in a cumulative manner at bath concentrations of 1 nmol/L, 10 nmol/L, 100 nmol/L, 1 μmol/L and 10 μmol/L at 20 minute intervals. A simultaneous control was also run which was exposed to PSS alone. The effects of each agonist were then assessed using the Macintosh Powerlab hardware package and Chart 4.0 software, by calculation of the mean amplitude of vascular tone which was converted to tension, expressed in grams calculated during 20-minute intervals after each individual drug addition.

### Drugs and solutions

5-HT and PgF2_α _were purchased from Sigma-Aldrich (Dublin, Ireland). A stock solution (10 ml) of 10 μmol/L 5-HT was made up deionised water and stored at 4°C. Serial dilutions were made up in deionised water on the day of experimentation.

A 10 μmol/L stock solution (1 ml) of PgF2_α _was made up in ethanol and stored at -20°C. Serial dilutions were made up in ethanol on the day of experimentation. Fresh Krebs – Henseleit Physiological Salt Solution was made and buffered daily. KCl solutions were prepared on the day of experimentation.

### Statistical analysis

The measurements of the contractile effects of 5-HT and PgF2_α_, expressed as tension in grams, for each bath concentration, were compared with the measurements obtained in control measurements, using ANOVA followed by a Tukey post-hoc test. Comparisons between obese and normal samples were made using a paired-sample t-test. A P value < 0.05 was accepted as statistically significant. The statistical package SPSS for Windows version 14.0 (SPSS Inc., Chicago, Ill, USA) was used for these statistical calculations.

## Results

**Figure 1:**
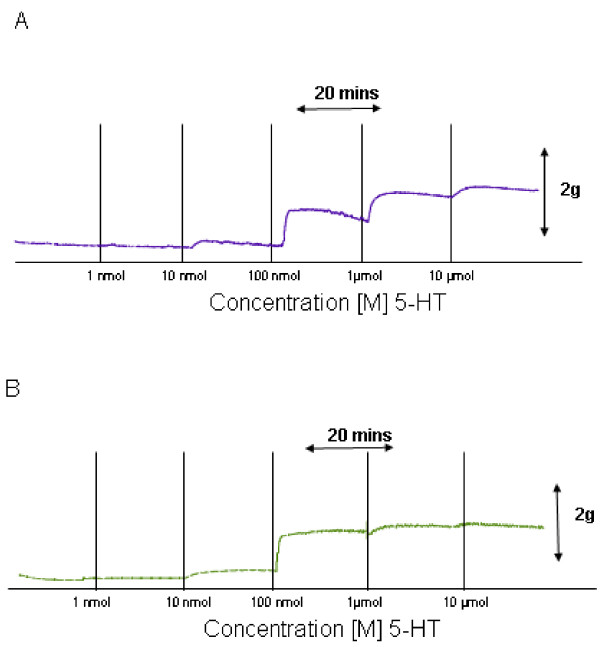
**Effects of 5 – Hydroxytrptamine (5-HT) on human umbilical artery tone**. Representative recordings demonstrating the vascular response elicited by cumulative additions of 5-HT [1 nmol – 10 μmol] at 20 minute intervals in an umbilical artery preparation from a woman of normal BMI (A), and that observed in an umbilical artery from an obese woman (B).

A representative recording demonstrating the vascular response elicited by 5-HT in an umbilical artery preparation from a woman of normal BMI is shown in Fig 1A. The mean maximum tension (MMT) ± standard error of the mean (SEM) observed was 2.97 ± 0.4 g (n = 5). It was observed that 5-HT concentrations of 100 nmol/L, 1 μmol/L, and 10 μmol/L exerted a statistically significant increase in vascular tone when compared to controls (n = 5; P < 0.05). Figure 1B shows a similar recording of raw data outlining the action of 5-HT on umbilical artery tone obtained from an obese woman. There was a resultant increase in tone at bath concentrations of 100 nmol/L, 1 μmol/L, and 10 μmol/L, when compared to control values (n = 5; P < 0.05). The MMT observed in preparations from obese women was 4.2532 ± 0.56 g of tension (n = 5). Although both groups demonstrated a similar contractile pattern the amplitude of the response was greater in the obese samples. Comparison of the amplitude measurements observed, at all bath concentrations, in the umbilical artery rings obtained from normal and obese women, revealed that arteries from obese women exhibited a greater contractile response than those from women of normal BMI (n = 5; P = 0.018). In Figure 3A a graph demonstrated the effects of 5-HT on umbilical arterial preparations from both groups. The values are demonstrated in Table 1.

**Figure 2:**
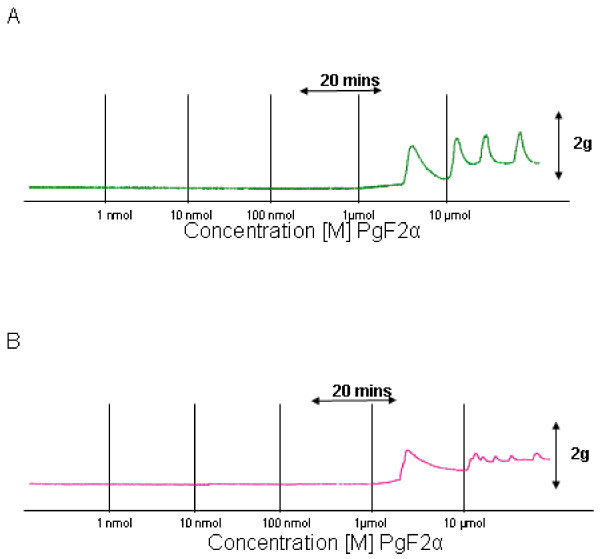
**Effects of Prostaglandin F-2α (PgF2α) on human umbilical artery tone**. Representative recordings demonstrating the vascular response elicited by cumulative additions of PgF2α [1 nmol – 10 μmol] at 20 minute intervals in an umbilical artery preparation from a woman of normal BMI (A), and that observed in an umbilical artery from an obese woman (B).

**Figure 3:**
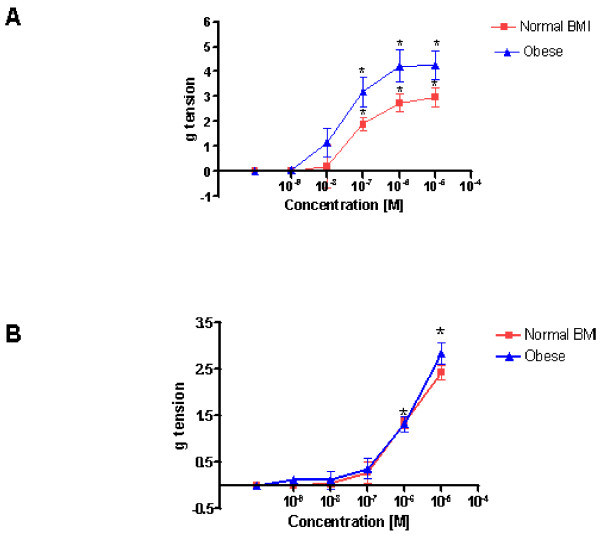
**A, The dose response curves demonstrate the increase in umbilical artery tone due to cumulative increasing tissue bath concentrations 5-HT [1 nmol – 10 μmol] on vessels from women of normal BMI (red closed squares) compared with vessels from obese women (blue closed triangles)**. Asterixes signify those concentrations at which test contractility seen in a test sample is statistically significant compared with a control (p < 0.05). **B**, The dose response curves demonstrate the increase in umbilical artery tone due to cumulative increasing tissue bath concentrations PgF2α [1 nmol – 10 μmol] on vessels from women of normal BMI (red closed squares) compared with vessels from obese women (blue closed triangles). Asterixes signify those concentrations at which test contractility seen in a test sample is statistically significant compared with a control (p < 0.05).

The vascular response elicited by PgF2_α _in an umbilical artery preparation from a woman of normal BMI is shown in Fig. 2A. At bath concentrations of 1 μmol/L and 10 μmol/L it exerted a statistically significant contractile effect in comparison to control experiments (n = 5; P < 0.001). The MMT measured was 2.358 ± 0.136 g of tension (n = 5). The effect of PgF2_α _on umbilical artery tone obtained from an obese woman is similarly shown in Fig. 2B and concentrations of 1 μmol/L and 10 μmol/L also exerted a statistically significant difference compared to controls. The MMT observed was 3.02 ± 0.251 g of tension (n = 5). There was a trend towards a greater contractile effect in obese arteries under the influence of PgF2_α _but this was not statistically significant (n = 5; P value = 0.358) the results are demonstrated graphically in Figure 3B.

**Table 1:** Effect of 5-HT and PGF-2α on human umbilical artery tone

Drug (Vessel Type)	N	MMC Effect (g tension)	P-Value (t-test)
5-HT (Normal)	5	2.97 ± 0.4	0.018
5-HT (Obese)	5	4.2532 ± 0.563	
PgF2_α _(Normal)	5	2.358 g ± 0.136	0.358
PgF2_α _(Obese)	5	3.02 g ± 0.251	

Values presented are means ± standard error of the mean (SEM).

## Discussion

There is good reason to hypothesize that the vascular reactivity of vessels from the feto-placental circulation, both large and small vessels, may be altered in association with obesity. Obesity is associated with alterations in both neuronal control, and peripheral secretion of peptides, all of which may alter vascular reactivity[5,13,14]. What is different for umbilical vessels is that the relative duration of exposure of these vessels in obese pregnant women to potential modulating factors, is short. 5-HT is a standard vasoactive agent used frequently in experiments investigating umbilical artery tone[15,16]. For this reason a major part of this study involved evaluating the effects of 5-HT on tone in vessels from women of normal BMI in comparison to tone in vessels from obese women. The criteria selected for a normal BMI, and for an obese BMI were strict, with the obese category designated to the morbidly obese range of BMI. The results for 5-HT clearly demonstrate a difference in the responsiveness, in terms of tone measurements, in umbilical artery specimens obtained from obese women. This increased vasoactive responsiveness in umbilical artery specimens from morbidly obese women, suggests that the factors modulating umbilical artery tone are different in these circumstances. As outlined previously, this may be due to alterations in regulatory peptide levels, or altered properties of the endothelium or muscularis layers[17,18]. While the signalling mechanisms that have mediated this are currently not clear, these experiments highlight the novel concept that regulation of feto-placental vessel flow may be altered in obese women with implications for fetal growth and wellbeing.

A similar model was used for PgF2_α_. The experiments were carried out in an exactly similar way. There was no significant difference in the contractile response of vessels taken from obese women in comparison to vessels that were obtained from women of normal BMI. It is difficult to make a reliable conclusion from the PgF2_α _experiments done in this study, other than the fact that PgF2_α _responsiveness is not altered in association with maternal obesity, as appears to also be the case with hypertensive disorders of pregnancy[19].

There are limitations to this study and issues raised by it that require further evaluation. While 5-HT is a very well established vasoactive agent in the umbilical arteries circulation, the role of PgF2_α _is not as clearly understood. Two vasoactive agents were investigated and hence it would be appropriate to test this hypothesis further using other compounds such as angiotensin and endothelin. In addition, while the changes outlined here in association with maternal obesity are clear, it would be most interesting to do myographic studies on small placental vessels to see if similar changes occurred. Finally, mechanistic experiments to evaluate the potential explanations for altered feto-placental vascular tone in association with obesity would be the next logical step to pursue.

In summary, these experiments highlight the novel concept, demonstrating that alterations in the contractile properties of feto-placental vessels may occur in association with maternal obesity. This raises the possibility that maternal obesity in pregnancy may have adverse effects on the feto-placental vasculature and hence have implications for fetal development and growth, as observed in other metabolic disorders.

## Competing interests

The authors declare that they have no competing interests.

## Authors' contributions

MPH carried out experiments and wrote the manuscript, ATM carried out experiments, SVG carried out experiments, JJM designed the study and wrote the manuscript. All authors have read and approved the final manuscript.
